# A randomized controlled trial of pre-conception treatment for periodontal disease to improve periodontal status during pregnancy and birth outcomes

**DOI:** 10.1186/1471-2393-13-228

**Published:** 2013-12-09

**Authors:** Hong Jiang, Xu Xiong, Yi Su, Yiming Zhang, Hongqiao Wu, Zhijun Jiang, Xu Qian

**Affiliations:** 1 School of Public Health, Fudan University, No. 138 Yixueyuan Road, Shanghai, China; 2 Key Laboratory of Public Health Safety, Ministry of Education, No. 138 Yixueyuan Road, Shanghai 200032, China; 3 School of Public Health and Tropical Medicine, Tulane University, 1440 Canal Street, Suite 2022, New Orleans, LA 70112, USA; 4 Eye & ENT Hospital of Fudan University, Shanghai, China; 5 Maternal and Child Health Care Hospital, No. 26 Boai Road, Changzhou Municipality, China; 6 Global Health Institute, Fudan University, Shanghai, China

**Keywords:** Pre-conception, Periodontal disease, Birth outcomes, Inflammatory mediators

## Abstract

**Background:**

Evidence has suggested that periodontal disease is associated with an increased risk of various adverse pregnancy and birth outcomes. However, several large clinical randomized controlled trials failed to demonstrate periodontal therapy during pregnancy reduced the incidence of adverse pregnancy and birth outcomes. It has been suggested that the pre-conception period may be an optimal period for periodontal disease treatment rather than during pregnancy. To date, no randomized controlled trial (RCT) has examined if treating periodontal disease before pregnancy reduces adverse birth outcomes. This study aims to examine if the pre-conception treatment of periodontal disease will lead to improved periodontal status during late pregnancy and subsequent birth outcomes.

**Methods/Design:**

A sample of 470 (235 in each arm of the study) pre-conception women who plan to conceive within one year and with periodontal disease will be recruited for the study. All participants will be randomly allocated to the intervention or control group. The intervention group will receive free therapy including dental scaling and root planning (the standard therapy), supragingival prophylaxis, and oral hygiene education. The control group will only receive supragingival prophylaxis and oral hygiene education. Women will be followed throughout their pregnancy and then to childbirth. The main outcomes include periodontal disease status in late pregnancy and birth outcomes measured such as mean birth weight (grams), and mean gestational age (weeks). Periodontal disease will be diagnosed through a dental examination by measuring probing depth, clinical attachment loss and percentage of bleeding on probing (BOP) between gestational age of 32 and 36 weeks. Local and systemic inflammatory mediators are also included as main outcomes.

**Discussion:**

This will be the first RCT to test whether treating periodontal disease among pre-conception women reduces periodontal disease during pregnancy and prevents adverse birth outcomes. If the effect of pre-pregnancy periodontal treatment is confirmed, this intervention could be recommended for application in low- or middle-income countries to improve both oral health and maternal and child health.

**Trial registration:**

This trial is registered with Chinese Clinical Trial Registry (ChiCTR): ChiCTR-TRC-12001913.

## Background

Periodontal disease is defined as an inflammatory condition of the soft tissues surrounding the teeth (i.e., gingivitis) and the destruction of the supporting structures of the teeth [1-3]. As a persistent bacterial infection, periodontal disease leads to a chronic and systemic challenge with bacterial substances and host-derived inflammatory mediators that are capable of initiating and promoting systemic diseases. There is increasing evidence suggesting that periodontal disease is associated with systemic diseases such as cardiovascular diseases, diabetes mellitus, as well as adverse pregnancy and birth outcomes [4-8].

### The relationship between periodontal disease and adverse birth outcomes

Since Offenbacher et al. first reported an association between periodontal disease and preterm birth in 1996 [9], substantial evidence has accumulated suggesting periodontal disease may be associated with an increased risk of various adverse pregnancy and birth outcomes such as preterm birth, low birth weight, early pregnancy loss, gestational diabetes and preeclampsia [10].

Inflammatory mediators induced by periodontal disease have downstream effects on biological pathways and tissues. Studies have shown increased levels of interleukin-1 beta (IL-1*β*), IL-6, tumor necrosis factor alpha (TNF-*α*, beta-glucuronidase (*β*–glucuronidase), prostaglandin E2 (PGE_2_), aspartate aminotransferase (AST), metalloproteinase-8 (MMPT-8) and decreased level of osteoprotegerin (OPG) not only in the gingival tissues, gingival cervicular fluid (GCF), saliva, but also in the serum/ plasma of patients affected by periodontal disease [2,11-19]. These inflammatory mediators appear in the systemic circulation and eventually cross the chorioamniotic barrier to finally appear in the amniotic fluid. Additionally, maternal periodontal disease results in placental and fetal exposure to microbes through hematogenous dissemination. Blood-borne bacterial products, especially lipopolysaccharide (LPS), target the chorioamniotic plexus to trigger local PGE and TNF-*α* synthesis. These host-derived inflammatory mediators urge preterm membrane rupture and labor, resulting in preterm delivery. This poor uteral environment could lead to fetal growth restriction (FGR) and neonatal morbidity.

### The optimal time for treating periodontal disease to improve pregnancy outcomes

Attempts at treating periodontal disease during pregnancy to achieve improved outcomes have had inconsistent conclusions. Recently, several large clinical randomized controlled trials failed to conclude that standard periodontal therapy during pregnancy reduced the incidence of adverse pregnancy outcomes (e.g., preterm birth and low birth weight) [20-22] The question of when to treat periodontal disease was put forward.

Pregnancy may not be an appropriate period for periodontal disease treatment as discussed by Xiong et al. [23]: 1) Treating periodontal disease during pregnancy may be too late to reduce the local and systemic inflammation activated by oral bacterial pathogens. 2) Dental scaling and root planning during treatment itself may cause bacteremia triggering systemic inflammation, leading to adverse pregnancy and birth outcomes, 3) Because of safety concerns on the frequency of periodontal treatment during pregnancy, the treatments are often restrict to 1 or 2 courses, which may not be effective in preventing the progression of periodontal disease.

In contrast, the pre-conception period may be optimal for interventions [23]: 1) Pre-conception periodontal therapy may lead to better outcomes as it can be more intensive compared with treatment during pregnancy (e.g., the use of adjunctive antibiotics). 2) Pre-conception treatments may provide more evidence as to whether periodontal disease is a causal risk factor for adverse pregnancy and birth outcomes (e.g. preterm birth et al.) 3) If pre-conception periodontal treatment is shown to be effective, it may highlight the biological mechanism of how subclinical infections such as periodontal disease lead to an increased risk of adverse pregnancy and birth outcomes. 4) Periodontal disease is preventable and curable. If the effect of pre-conception periodontal treatment is confirmed, this treatment will lead to improved pregnancy and birth outcomes worldwide. Therefore, periodontal treatment either before pregnancy (for nulliparous women) or in the period between pregnancies (for multiparous women) may reduce adverse pregnancy outcomes.

No randomized controlled trial (RCT) to date has examined if treating periodontal disease among pre-conception women (e.g., women who plan to conceive within one year) reduces the rate of adverse pregnancy or birth outcomes. Therefore, we propose to conduct this RCT of pre-conception treatment for periodontal disease to improve periodontal status during pregnancy and birth outcomes. In order to explore the biological mechanism of periodontal disease and how it leads to increased risk of adverse birth outcomes, we will also test and compare the changes in inflammatory mediators mentioned above in saliva, maternal blood and cord blood between the intervention and control groups.

### Objectives and hypotheses

The objective of the study is to examine if the pre-conception treatment of periodontal disease leads to the improved periodontal status during late pregnancy and birth outcomes (e.g. mean birth weight and mean gestational age).

We hypothesize that the pre-conception treatment of periodontal disease (periodontal treatment one year before pregnancy) will:

● Reduce rate of periodontal disease during pregnancy.

● Increase birth weight and prolong gestational duration.

● Reduce mean levels of local and systematic inflammatory mediators.

The primary outcome is periodontal disease determined by measuring probing depth (PD), clinical attachment loss (CAL) and percentage of bleeding on probing (BOP) between 32 and 36 weeks of gestational age. Secondary outcomes are mean birth weight (grams) and mean gestational age (weeks). In addition, we will compare inflammatory mediators (i.e. IL-1β, IL-6, β-glucuronidase, TNF-α, PGE_2,_ AST, MMP-8, OPG) in saliva, as well as in maternal blood during late pregnancy and cord blood after delivery, in both groups as secondary outcomes.

## Methods/design

### Overall study design

**Figure 1:**
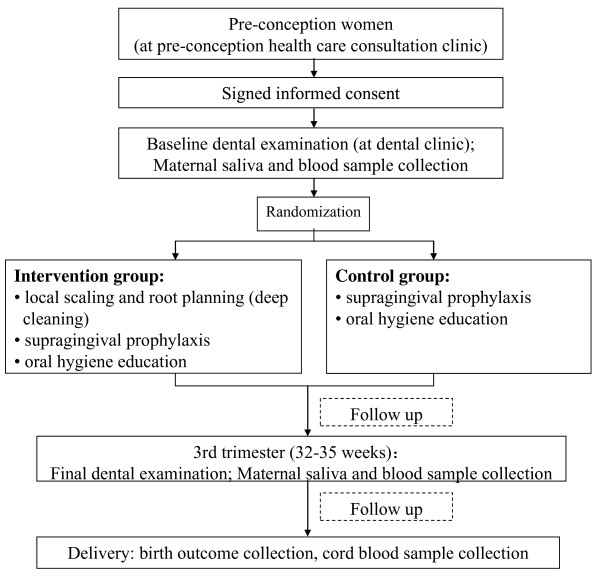
Research flow chart.

The design of this proposed study will be a randomized controlled trial in which pre-conception women who plan to conceive within one year will be eligible to participate in an oral examination. Women who meet the criteria for periodontal disease will then be randomized into two groups: an intervention or periodontal treatment group, versus a control group. All participants who get pregnant within one year will be followed throughout their pregnancy (see Figure 1). Ethical approval to conduct the trial has been granted by the Institutional Review Board of the School of Public Health, Fudan University, Shanghai, China (IRB# 2011-03-0271) and the study is registered with Chinese Clinical Trial Registry (#ChiCTR-TRC-12001913).

### Participants and recruitment

This RCT will be conducted at the Maternal and Child Health Care Hospital, in Changzhou Municipality, China. This hospital was chosen because it contained both pre-conception consultation and dental clinics. Changzhou is a medium sized city in eastern China, with a population of 4,600,000 (in 2012). The hospital is the only maternal and child health care hospital in Changzhou Municipality, with a total of 9,000 deliveries annually (covering more than 70% of deliveries in the city).

Enrollment will be conducted among women attending pre-conception health care counseling at the hospital. All women attending pre-conception consultation clinics will be approached by research assistants for their willingness to participate in the study, and then will be assessed for their eligibility to participate. Eligible women will be invited to participate in a free full-mouth dental examination to screen for periodontal disease. Women who meet the criteria for having periodontal disease will be invited to participate in this trial and be randomized into the intervention and control groups. Written informed consent will be obtained from each participant before the randomized group allocation is revealed.

### Inclusion criteria

● Pre-conception women planning to conceive within one year.

● At least 18 years old.

● Planning to deliver at the recruiting hospital.

Additional inclusion criteria for randomization:

● Diagnosed as having periodontal disease after a dental examination.

● At least 20 teeth.

● Without moderate or severe dental caries.

● Without systemic or reproductive disease.

● Without rheumatoid arthritis.

● Without severe cardiovascular disease, diabetes, hyperthyroidism, and/or other systemic diseases.

● Without immunodeficiency diseases.

● Willing to be compliant to follow up during the trial.

### Exclusion criteria

● Less than 18 years old.

● Fewer than 20 teeth.

● Contraindication to probing in a dental examination and treatment, such as heart disorders.

● Unwilling or unable to sign the informed consent form.

● Receiving periodontal treatment within the past six months.

● Changing pregnancy plan (no plan to be pregnant within a year).

● With a disease mentioned in the inclusion criteria.

### Sample size

#### Sample size calculation for primary outcomes

We calculated that a consecutive sample of 470 (235 in each arm of the study) pre-conception women with periodontal disease will be required for the study. The sample size calculation is based on detecting changes in primary outcome of periodontal disease measurements- probing depth (PD) and clinical attachment loss (CAL) in millimeters (mm). To detect a difference in mean PD of 0.15 mm between the groups in the 3^rd^ trimester, a sample size of 141 for per group is needed. This was based on an estimated mean PD of 1.6 mm and SD of 0.2 mm from the earlier pilot study of 53 pre-conception women (mean PD of cases = 2.0 mm, SD = 0.3 mm; mean PD of non-cases = 1.8 mm, SD = 0.4 mm). After allowing for an estimated 25% exclusion due to non-pregnancy at the end of the first year and 20% loss to follow up during pregnancy, we will recruit a total of 470 pre-conception women with periodontal disease. With the estimation of 55% prevalence of periodontal disease (from the pilot study), 854 pre-conception women will be recruited for screening periodontal disease to identify 470 women with periodontal disease. The sample size calculation based on CAL is the same as the calculation using PD.

#### Calculation of statistical power for secondary outcomes

The power calculations for secondary outcomes include mean birth weight, mean gestational age, and levels of selected inflammatory mediators in the 3^rd^ trimester.

##### Mean birth weight

Given the initial sample size of 141 in each group for detecting differences in PD and CAL measurements between the intervention and control groups, the proposed study will have 99.7% statistical power to detect a difference in birth weight of 200 g, with a SD 400 g and 300 g in each group respectively, at 0.05 significance level.

##### Mean gestational week

Given the sample size of 141 in each group, the proposed study will have 86.9% statistical power to detect a difference in gestational week of 0.5 week between two groups, with SD 1.5 week and 1.2 week in each group respectively, at 0.05 significance level.

##### Selected mean biological markers

Given the sample size of 141 in each group, the proposed study will have statistical power ranging from 89.2% to 99.9% to detect differences in the selected mean biological markers including AST, TNF-α, IL-1β, IL-6, MMP-8 at 0.05 significance level [18,24-26].

### Enrollment and randomization procedures

Random allocation to either the intervention or control group is concealed by sequentially numbered, sealed opaque envelopes containing the group allocation, which will be determined by a computer generated random number. A research group member who has no direct contact with participants will be responsible for generating the random numbers and preparing the envelopes. Immediately after the dental examination, the nurse will open the sealed envelope and inform the participant of the group allocation.

### Intervention group

Women in the intervention group will be provided with free treatment for periodontal disease. The periodontal therapy includes local scaling and root planning (standard therapy), supragingival prophylaxis, plus oral hygiene education (e.g., tooth brushing instructions). After the initial treatment, women in the intervention group will be requested to have re-examination and re-treatment every three months until they become pregnant. All of the examination, treatment and health education will be free. Once women are identified as pregnant, treatment will be stopped.

### Control group

Women in the control group will not receive the same local therapy but will instead receive supragingival prophylaxis, plus oral hygiene education. They will be advised to have re-examination every three months in the following year. In the 3rd trimester, women in the control group will be asked to have a periodontal examination.

### Data collection

#### Baseline data

Prior to the periodontal examination, a questionnaire will be administered to collect information such as demographics, socio-economic status, medical history, and use of dental care, etc.

#### Periodontal disease measurements

The initial pre-conception periodontal examination will be recorded and will reflect the baseline periodontal status. Measurements will be taken at six sites per tooth (Mesio-buccal, mesio-lingual, disto-buccal, disto-lingual, mid-buccal and mid-lingual), using a manual UNC-15 probe [3]. There is no universally accepted research definition for the diagnosis of periodontal disease. In previous studies, definitions that combined PD and CAL over a certain threshold were used [3,10]. PD was defined as the distance in millimeters from the gingival margin to the apical part of the pocket. CAL represents the distance between the cemento-enamel junction (CEJ) and the base of the pocket. CAL is obtained by adding the PD measurement to the recession that is the distance between the CEJ and the gingival margin. In this study, periodontal disease was defined as a presence of any sites exhibiting PD < 3 mm or CAL < 3 mm.

All periodontal measurements will be performed by one dentist who will be blinded to the group allocation of pre-conception women to ensure study reliability. Another dentist will be responsible for periodontal therapy, supragingival prophylaxis and oral health education. A dental nurse will record the scores on clinical periodontal indexes including PD, BOP and recession of each site for each woman during the oral examination.

Prior to the enrollment of patients in the study, dentists will be invited to participate in a calibration study. During this section, three healthy volunteers will be recruited and will be examined by dentists and an experienced periodontist. Inter- and intra-examiner variations in periodontal probing measurements (e.g., PD, recession) will be identified between and among dentists and the experienced periodontist. They will discuss the difference and adjust the measurement approach until a final agreement is reached. After calibration, intra- and inter-examiners’ agreement rates of PD measurements within ± 1 mm should be reached. Kappa statistic and intra-class correlation coefficient among dental examiners for the studies will be calculated.

The last dental examination will be carried out in the 3^rd^ trimester as the final evaluation data.

#### Biologic sample collection

##### Saliva collection

We choose saliva samples to reflect periodontal health instead of gingival cervicular fluid (GCF) due to the following advantages: (1) Compared with GCF sampling, saliva sampling is convenient and simple. (2) The concentration of salivary components from the analysis reflects progression and severity of periodontal disease. (3) The level of the ingredients in saliva may provide evidence for the need for further treatment and the effect of treatment response. (4) Saliva reflects the overall periodontal health of the patient, rather than at a point [27,28].

In this study, saliva will be collected before the initial pre-conception dental examination and prior to the last examination in the 3^rd^ trimester. We collected whole expectorated saliva, without stimulation, from each participant using an adapted version of the method described by Miller et al. [2]. Women will be asked to rinse their mouths with tap water, wait for 5 minutes, and then expectorate whole saliva into sterile tubes. The samples will be immediately centrifuged, and the upper clear portion will be kept and frozen at −80°C.

##### Blood sample collection

During women’s routine blood draws at both their pre-conception health care visit and their antenatal visit in the 3^rd^ trimester, 5 ml extra blood will be collected from each woman and kept at −80°C for the proposed study.

##### Cord sample collection

Immediately after delivery, 5 ml cord blood will be collected and kept in −80°C.

##### Birth outcomes

Reviews of medical records will be conducted by the research nurse to record birth weight and gestational age. Pregnancy history, birth outcomes, as well as adverse events will be documented using a uniformed form.

### Data management

Given the nature of the intervention, participants will not be blinded to their group allocation. To minimize bias, the dentist who is going to perform the baseline and final periodontal examination will be blinded. All treatments and oral health care will be carried out by another dentist. Furthermore, researchers who collect biological samples will be blinded to the allocation of women’s group.

All materials containing individual information of participants will be stored in a locked cabinet and only accessible to research team members. The computer with research information and data will be password protected and only be accessed by authorized research team members.

### Data analysis plan

All analyses will be conducted with “intention to treat” analysis [29]. Descriptive statistics will be performed to examine for all exposures, outcomes and covariates. For continuous variables, such as PD, CAL, birth weight (grams), gestational age (weeks), biologic parameters, means will be compared using t-tests, or non-parametric equivalents for non-normally distributed variables. For categorical variables such as the rate of periodontal disease, chi-squared tests will be used. The outcomes will be compared between the intervention and control groups, and between pregnancy and pre-conception groups.

### Data safety monitoring plan

To ensure the safety of research participants and the validity and integrity of data, we will develop a Data Safety Monitoring Plan and establish an independent Data Safety and Monitoring Board (DSMB). The primary responsibilities of the DSMB are to develop protocol stopping guidelines related to the safety of individuals and the overall trial.

## Discussion

To our knowledge, this is the first study developed to test the efficacy of pre-conception periodontal disease treatment in the prevention of adverse birth outcomes. The pre-conception periodontal treatment mainly includes scaling and root planning (standard therapy) in combination with personal oral hygiene. These are non-surgical periodontal procedures and can be performed by oral health professionals (e.g., dentists or dental hygienists) after receiving appropriate training. In addition, since periodontal treatment is performed before pregnancy, it will avoid potential risks to the pregnancy (fetus) and will be less stressful to women. If the intervention is effective, it will be more acceptable by women and society, and have potential generalizability. Furthermore, the study will help develop a model of pre-conception oral health care to improve both oral health and maternal and child health nationally and internationally. The pre-conception periodontal treatment strategy may be applicable to many low-or middle-income countries (e.g., India and China), where childbearing aged and pregnant women have no or limited access to preventive or restorative dental care.

Due to the limited funding resource, the sample size of this study is relatively small, which may not allow for the observation of some pregnancy outcomes e.g. rate of preterm birth and low weight birth, rate of gestational diabetes, rate of preeclampsia, and other birth outcomes. If there is evidence of effectiveness after this initial study, we will seek further funding to expand the trial, with pregnancy and birth outcomes (e.g. rate of low birth weight and preterm birth) as primary outcomes.

## Abbreviations

AST: Aspartate aminotransferase; BOP: Bleeding on probing; β–glucuronidase: Beta-glucuronidase; CEJ: Cemento-enamel junction; CAL: Clinical periodontal loss; DSMB: Data Safety and Monitoring Board; FGR: Fetal growth restriction; GCF: Gingival crevicular fluid; IL-1β: Interleukin-1 beta; IL-6: Interleukin-6; LPS: Lipopolysaccharide; MMPT-8: Metalloproteinase-8; OPG: Osteoprotegerin; PD: Probing depth; RCT: Randomized Controlled Trial; TNF-α: Tumor necrosis factor alpha.

## Competing interests

The authors have no conflicts of interest to disclose.

## Authors’ contributions

XX, QX, JH and SY conceptualized and designed the study, contributed to the development of the trial and obtaining the funding. ZYM, WHQ, JZJ contributed to the study design. JH and XX drafted the manuscript. All authors provided critical comments on intellectual contents and have approved final text.

## Authors’ information

Xu Xiong is co-first author.

## Pre-publication history

The pre-publication history for this paper can be accessed here:

http://www.biomedcentral.com/1471-2393/13/228/prepub
